# Threats of contamination of drinking water sources and their protection in the case study of the Czech Republic

**DOI:** 10.1007/s11356-025-36542-w

**Published:** 2025-05-29

**Authors:** Štěpán Kavan, Šárka Kročová, Milan Axman, Eva Stýblová

**Affiliations:** 1 Faculty of Health and Social Studies, University of South Bohemia in Ceske Budejovice, 370 04 Ceske Budejovice, Czech Republic; 2 Faculty of Safety Engineering, VSB‐Technical University of Ostrava, Ostrava, 700 30 Czech Republic

**Keywords:** Aquatic ecosystem, Contamination, Drinking water source, Risk analysis methods, Risks definition threat elimination, Pollution, Water supply system

## Abstract

The permanent protection of groundwater and surface sources of drinking water in the required raw water quality from contamination is the primary foundation of preserving the life of society and the entire engineering infrastructure operability not only in the Czech Republic but also in other countries of the world. One of the basic ways of contamination prevention enhancement and risk mitigation of putting a water source out of operation is the identification of the given risk as well as soil contamination monitoring. The purpose of this study is to understand specific aspects of contamination threats to drinking water sources and determine possible solutions to eliminate the risk, shown in the example case study in the Czech Republic conditions. Based on the findings, a proposal to enhance the prevention of water source contamination has been elaborated in the form of recommendations regarding the ways and means used to monitor the risk areas and to implement technical measures so as to eliminate or terminate the specified risk. A complex approach based on the integration of interdisciplinary factors and methodology is required to minimize the threats of aquatic ecosystems contamination in the Czech Republic.

## Introduction

Aquatic ecosystems are indisputably the most vulnerable infrastructure of every nation in the world. Their high-level vulnerability stems from their root state as they exist in the environment, which is affected by climatic properties, their intensity, and in recent centuries also by anthropogenic factors caused by human activities (Bozek et al. 2014; Ivančík and Nečas 2017). If one has no means and no influence on the course of natural events in the climate sector, they can at least carry out an analysis of these events using suitable mathematical methods (Bross and Krause 2017; Krajewska et al. 2021) and take necessary measures to mitigate the negative impacts on aquatic ecosystems, at the minimum in the following areas:

- Treating/handling rainwater
- Artificial retention of surface water in the landscape
- Protection of groundwater and surface water from contamination
- Monitoring contamination and risk assessment
- Solution of contamination using progressive engineering methods
- Maintaining long-term sustainability of aquatic ecosystems in a state of positive balance

Achieving the above-mentioned state is economically expensive especially in industrial zones and regions where the water contamination is currently beyond the threshold. It is vital to intensively deal with environmental quality of agroecosystems by means of monitoring individual detected outputs (Hajkowicz and Higgins 2008). Attention is to be drawn to the increasing threat of contamination caused by plastics and compounds, which disrupt the endocrine system, by heavy metals in water and by other biological and chemical substances used in the engineering infrastructure of the given region (Hashemi et al. 2013; Kováčová and Vacková 2014). The Czech Republic is well-known for not owning a significant source of flowing water for water resource management purposes nor a source suitable for power supply and other technical and operational uses. The country is entirely dependent on snow and rain precipitations and their intensity.

Adverse processes that limit, pollute, and transform water resources can generally be either natural or anthropogenic, or a combination of both (Quevauviller 2010, 2011; Sivakumar 2011; Garnier et al. 2015). These serious causes include processes that negatively affect climate change by their behavior, such as the tilt of the Earth’s axis, the intensity of solar radiation, changes in plate tectonics and volcanic eruptions, increasing concentrations of greenhouse gasses, and the shrinking of land, shelf, and mountain glaciers, sea level rise, etc. (Stocker et al. 2013), and secondarily, they can cause pollution of water resources, spatial and temporal distribution of water resources. As a result, water resources are not evenly distributed around the world (Richey et al. 2015). For example, purely human causes include a growing world population, which brings with it an increasing rate of urbanization (United Nations 2014) and an increasing number of developed areas around the world. Two negative trends are closely related to these causes. The first is called Urban Sprawl (Eigenbrod et al. 2011), when commercial, logistic, industrial, and other enterprises spread over the cities on the so-called green field, and the second is called Urban Sealing, where all types of buildings lead to the transformation of natural permeable surfaces into impermeable surfaces (Zarghami and Szidarovszky 2009). Last but not least, we must also include in this list the growing commercial deforestation and global pollution of the environment, with which the pollution of the world’s oceans (Lützhøft et al. 2012; Halpern et al. 2015) and rivers (Greenpeace International 2011; Pistocchi et al. 2012; Lofrano et al. 2015). These can be the only source of drinking water in many countries and, if polluted, can limit the life of the local society.

Within the scope of the topic addressed, different approaches in practice and their reflection can be seen. As part of the overview and introduction to the topic, only some selected aspects and reflections can be mentioned. The water quality and its seasonal changes in the Gomal Zam Dam and tributaries in the South Waziristan district of Pakistan were addressed using a scientific approach and investigation. The water samples that were taken were subsequently analyzed and evaluated within the framework of the drinking water guidelines set by the World Health Organization, with the exception of turbidity. The water characteristics were evaluated in terms of the water quality index and sodium hazard. The winter season had slightly worse water quality compared to the summer season due to higher contamination. Statistical analyses revealed that geogenic sources of rock weathering are the dominant factor for controlling the water quality of the area (Muhammad and Ullah 2022). A similar topic was also addressed in a study that examined heavy metal concentrations in water and sediment of the Kunhar River and its tributaries in the Kaghan Valley in northern Pakistan (Muhammad et al. 2021).

Climatic changes may increase the risk of aquatic ecosystems contamination in various ways (Intrieri et al. 2020). Some of the main threats of aquatic ecosystems contamination in new climatic conditions include the following: 

### Precipitation Changes

Extreme precipitations may lead to floods, which wash polluting substances down from the landscapes into water bodies and reservoirs. On the contrary, a long-term drought may cause accumulation of polluting substances in surface waters as the precipitation shortage decreases waterflow, which slows down their diluting and washing away.

### Rising Water Temperatures

Higher water temperatures may increase the risk of toxic algae and cyanobacteria growth, which produce toxins known as cyanotoxins. These substances may threaten the health of aquatic organisms as well as people that come in contact with them.

### Changes in the Volume of Glaciers and Snow

Melting glaciers and snow may release pollutants accumulated over the long-term such as heavy metals and persistent organic substances, which may contaminate bodies of water and reservoirs.

### Increased Frequency of Extreme Events

Increased frequency of extreme events, such as storms and floods, may cause the release of hazardous chemicals from industrial facilities and warehouses into aquatic ecosystems.

### Acidity of Oceans

Ocean acidification may influence chemical processes which affect the availability and toxicity of certain substances in aquatic ecosystems. This may have an indirect impact on the health of aquatic organisms.

### Changes in Soil Use

Changes in the use of soil as a result of climatic changes such as agricultural expansion or mining may increase the risk of pesticides, fertilizers, and other pollutants seeping out into aquatic ecosystems.

Scientific literature is concerned with ecosystem improvement. Authors define how the use of sustainable procedures has led to the increase of implementation of green technologies and nature-friendly solutions (Hálová & Alina 2014). Based on the research, the range of options used to improve the environmental health of urban spaces is being solved. The analysis revealed that cases using an integrated approach to the solution show high ambitions. Nature-friendly solutions, especially green technologies, appear to be attractive tools which might help improve the current situation (Addo-Bankas et al. 2022). Research, which proved that nature-friendly measures mitigate and solve the depletion of sources and climate problems, came to similar results (Oral et al. 2021).

Other topics considered include rainwater management and a focus on urban resistance including international relations (Kazansky 2022; Ivančík and Andrassy 2023). Attention is paid to the scope of resilience of urban rainwater drainage systems, which the authors describe. There are other solutions for managing rainwater (Barreiro et al. 2024). Significant parts of water sources have been polluted in recent years, especially those near cities and industrial agglomerations. Therefore, the monitoring of water bodies and the subsequent assessment of possible risks rises in importance. This problem is being solved and, simultaneously, the need of using new and economically more effective monitoring methods used to check the decrease of polluting sources is emphasized (Naimaee et al. 2024). A major segment is the sustainability of aquatic ecosystems, where tools, indicators, and approaches currently used in the field of aquatic ecosystems and their potential contamination play an important role (Odume and Wet 2019). An integral part is the assessment of ecosystem services, which is gaining much more importance in sustainable and ecological development (Wei & Zhan 2019).

The article “Water, Resources, and Resilience: Insights from Diverse Environmental Studies” brings together a rich tapestry of research contributions that collectively emphasize the paramount importance of water management and sustainability. As our global community faces escalating water challenges driven by climate change, population growth, and pollution, the need for innovative solutions and informed decision-making becomes increasingly urgent (Pietrucha-Urbanik and Rak 2023). Another view and approach is the application of selected analytical methods in order to analyze field data and subsequently perform their interpretation. Selected methods like regression analysis and fuzzy inference system are used for the data assessment (Vališ et al 2014).

Another perspective is the investigation of the physicochemical parameters of water and sediments in the Naltar lakes in northern Pakistan. The water and sediments were analyzed for physicochemical parameters. The water quality parameters in the research were evaluated for drinking and irrigation water quality indices. Heavy metal concentrations in the sediments showed a moderate level of contamination, which poses a low risk to the lake ecosystem (Muhammad 2023). Another study similarly focused on determining the contamination of potentially toxic elements in the water of the alpine lakes of Gilgit-Baltistan in northern Pakistan. Water samples were collected from different alpine lakes and analyzed for the determination of potentially toxic elements using the atomic absorption spectroscopy method (Ullah and Muhammad 2024).

The study focused on water quality, which investigated the groundwater quality of various water sources for drinking and irrigation purposes in Hangu District, Khyber Pakhtunkhwa, Pakistan. Groundwater samples for the research were collected from various sources, including spring, bore well, dug well, and tube well, and analyzed for physicochemical parameters. The results showed that most of the physicochemical parameters were found to be within the World Health Organization (WHO) guidelines set for drinking water (Din et al. 2023).

The study responds to a certain incoherence and inadequacy of the perception of the risks of pollution of drinking water sources and an isolated approach to the prevention of their protection in the conditions of the Czech Republic. Climate change, the necessity of changing the approach to rainwater management, and the risk of pollution of drinking water sources are essential topics for sustainable living. The purpose of this study is to understand specific aspects of contamination threats of drinking water sources and determine possible solutions in order to eliminate the risk, shown in the example case study in the Czech Republic conditions.

Research questions were stated as follows: 

- What is the approach when handling snow and rain precipitations?
- How is the surface and groundwater protection against contamination implemented?
- What is the approach regarding contamination prevention and maintaining a long-term sustainability of aquatic ecosystems?

## Materials and methods

Several major threats of aquatic ecosystem contamination may be identified in the Czech Republic. These threats are associated with various human activities and environmental factors.

An overview of the threats is described in Table 1.

**Table 1:** Overview of possible threats of aquatic ecosystem contamination in the Czech Republic according to the authors

	Threat	Threat characteristics
1	Agricultural pollution	Although agriculture is an important economic sector in the Czech Republic, the use of fertilizers, pesticides and chemicals may lead to them seeping into rivers and groundwater. This may cause contamination of aquatic ecosystems and have a negative impact on biodiversity
2	Industrial pollution	A great number of industrial branches in the Czech Republic release different chemicals and toxic substances into rivers and surface water. This type of pollution may carry serious consequences for aquatic ecosystems and people dependent on these water sources
3	Sewage water	Insufficient or ineffective treatment of communal waste water may lead to the release of pollutants, including organic substances, nitrogen compounds and phosphorus into bodies of water and reservoirs
4	Surface runoff and erosion	Landscape changes caused by human activity, such as deforestation, agriculture and urbanization may increase surface runoff and erosion, which may contribute to sediments, nutrients and toxic substances seeping into aquatic ecosystems
5	Oil leakage	Oil spills released from ships, pump stations, chemical plants and oil refineries may pollute water sources and have a catastrophic impact on aquatic ecosystems and coastal areas
6	Pollution resulting from atmospheric emissions	Pollutant emissions into the atmosphere may enter aquatic ecosystems through precipitations and atmospheric depositions, which may lead to contamination of water sources
7	Algae bloom	Some types of algae can produce toxins such as microcystins and saxitoxins. These substances can be harmful to human and animal health. The presence of large amounts of algae can cause blockage of filtration systems in water treatment plants, which can reduce the efficiency of the water treatment process and increase maintenance costs

The study is written in the form of a case study using a detailed research approach aimed at the examination of a particular case—contamination threats of drinking water sources and prevention enhancement of their protection under Czech conditions. In the case study, the particular cases and pollution threat conditions are analyzed from different points of view. The case study is compiled based on the collection and analysis of data. The aim of the case study is to understand specific aspects of contamination threats of drinking water sources, prevention enhancement of their protection under Czech conditions, and to gain deeper knowledge which might be used to solve problems or to develop theoretical concepts. The case study is used to illustrate particular principles, strategies, and approaches in practice and works as a useful tool for the demonstration of particular concepts and approaches.

FMEA—Failure Mode and Effects Analysis method serves partially as a support tool. It is an analytical method focusing on the identification of possible defect occurrence points. FMEA method is to be treated as a living document, which needs to be updated regularly. The risks and the efficiency of determined measures of their elimination are to be assessed regularly as well (Subriadi and Najwa 2020).

In order to meet the objective and to develop the basis for the research question, a systematic approach of literature search was used to obtain current information sources, published results, and information in the field of threats of pollution of drinking water sources and prevention of increasing their protection. Furthermore, the method of analysis and synthesis was used, i.e., breaking down the whole into sub-components and combining the individual information into a whole, describing the principles in interdependencies. The above procedure was used in the analysis of the actual information and especially in its synthesis in the final part of the research. Another method used to elaborate the research objective was deduction, a process of reasoning from premises, where a conclusion is reached by proof. The procedure was applied in the elaboration of the findings of the empirical investigation into the summary final part of the research.

## Results

### Handling snow and rain precipitations

**Fig. 1:**
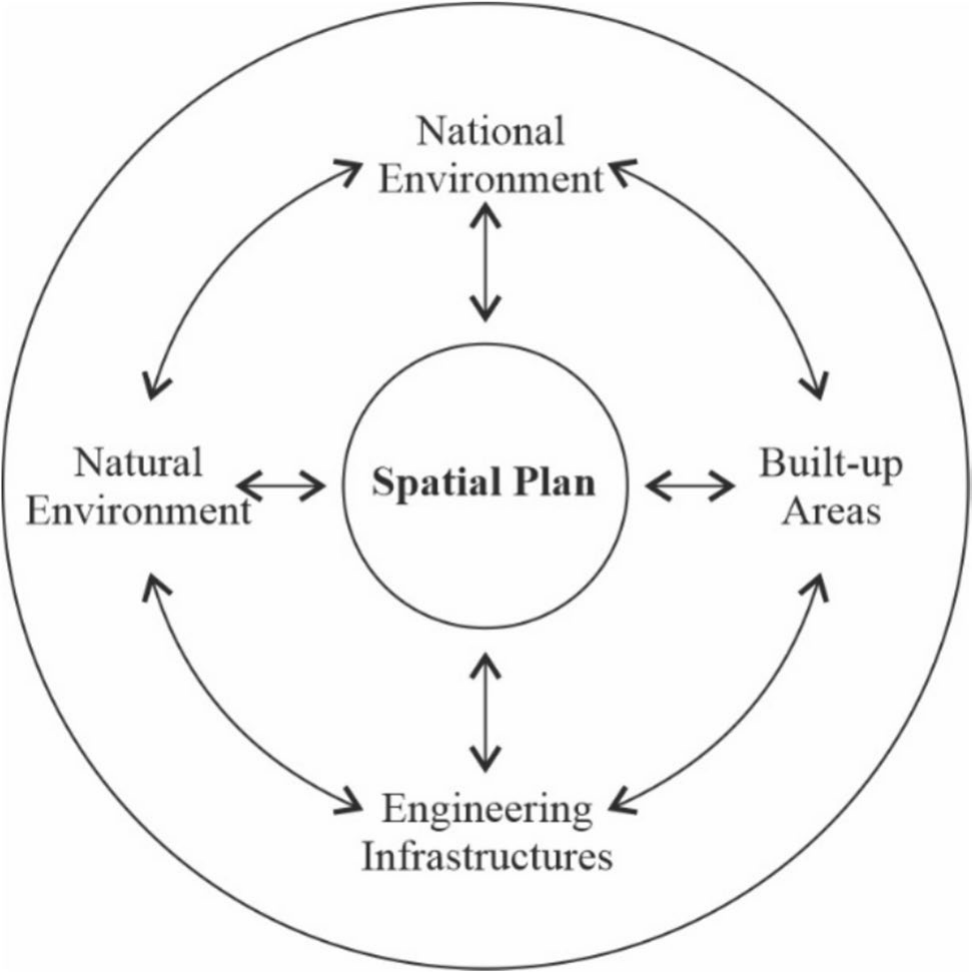
Diagram showing the influence of a spatial plan on the sustainability of the environment of the Czech Republic/authors/

A spatial plan, see Fig. 1, carries an exceptional importance in terms of a long-term sustainability of aquatic ecosystem function under set and constant conditions.

A spatial plan, developed optimally and for a long-term scope, is the prime protection of a region from alternative negative secondary impacts of water, soil, air pollution and other influences on the health of people, fauna, and flora caused by the burden of technological development (Loukas et al. 2007). With the relatively small size of the Czech Republic, its high traffic and engineering infrastructure density, and a high number of logistics and business zones, the spatial plan is to fulfill the regulation role in terms of preserving enough free space for the long-term sustainability of the unspoilt natural environment with minimal influences and risks of contamination by undesirable substances, including the precipitation intensity and occurrence in individual regions of the Czech Republic and the risk analysis after a significant change, as a consequence of climatic change in Central Europe.

While assessing the risks resulting from a potential lack of drinking water in the Czech Republic or any other region, in order to optimize the process, it is desirable to carry out a balance sheet regarding the total water supply in the site of origin, time imbalance, and their continual renewal in terms of the Earth’s water cycle in the primary part of the analytical analysis. It is crucial to keep in mind that the Czech Republic is 100% reliant on rainwater. The goal of every water manager has to be an effort made to retain the surface runoff of the Czech region for the longest time period possible so that it improves the water infiltration process and thus strengthens the groundwater repositories. The water management tendencies in the second half of the twentieth century were exactly contrary. A nationwide regulation of recipients with the aim of quick water drainage from the Czech Republic was taking place regardless of the fact that the area is a geographical “roof of Europe.” Therefore, it is a strategic necessity to retain water in water reservoirs, especially in the relief, to the utmost extent.

Precipitations are the product of condensation or desublimation of water vapor in the air or on the land surface. The average precipitation height in a drainage basin is expressed by the thickness of the water layer from rainfall per a set time period. It is expressed in mm and is defined as follows (Quevauviller 2011):

$${\overline H}_s=k\cdot\frac SF$$

where.

${\overline H}_s$—average precipitation height in a drainage basin [mm].

*F*—surface area of the drainage basin [km^2^].

*k* = 10^−3^—coefficient.

*S*—total precipitation volume [m^3^].

Because the Czech Republic has no other continual source of water renewal but rainwater, it is important to minimize the range of its direct surface runoff and thus achieve a point where the runoff occurs mainly in rain-free periods. As groundwater is recharged by the process of infiltration, it is appropriate to make favorable conditions in built-up areas by either natural or artificial infiltration even at increased investment costs.

**Fig. 2:**
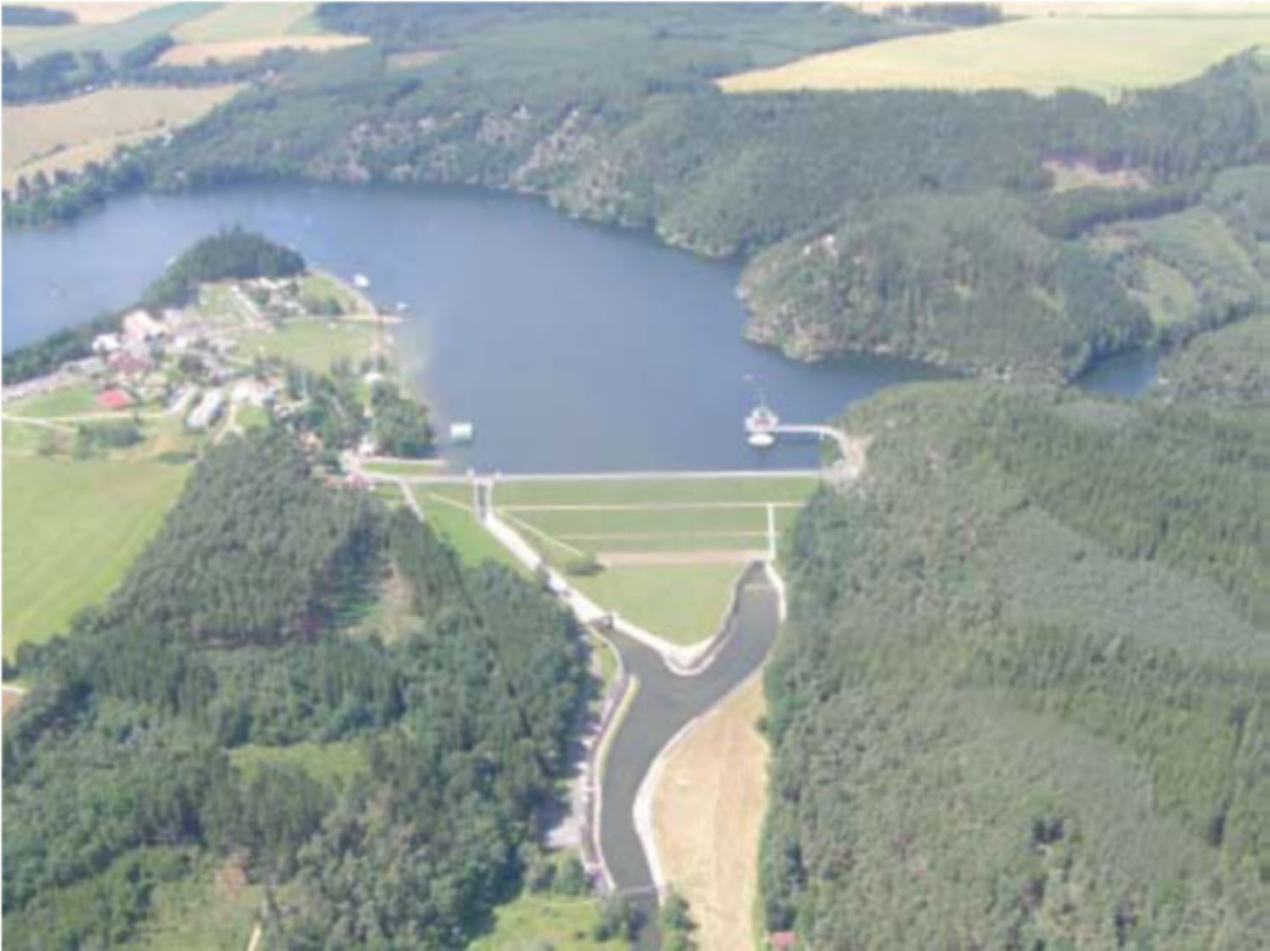
Water reservoir/authors/

As shown in Fig. 2, a spatial plan in a water management area creates conditions for the construction of artificial surface water reservoirs, which may be used secondarily for supplying the population with drinking water even in regions lacking enough groundwater for given purposes.

Due to the relatively fast-changing climatic conditions in the world, the primary natural water cycle will no longer be sufficient in relation to the needs of the engineering infrastructure of individual countries. A number of regions have already been affected by recurrent meteorological and subsequent hydrological droughts. Therefore, it will be crucial to take a wide range of costly precautionary measures in the field of aquatic ecosystems.

Some of the measures might be the steps listed below: 

1. Artificial water infiltration into aquiferous layers
2. Artificial infiltration of surface water into groundwater
3. Construction of new water reservoirs

**Fig. 3:**
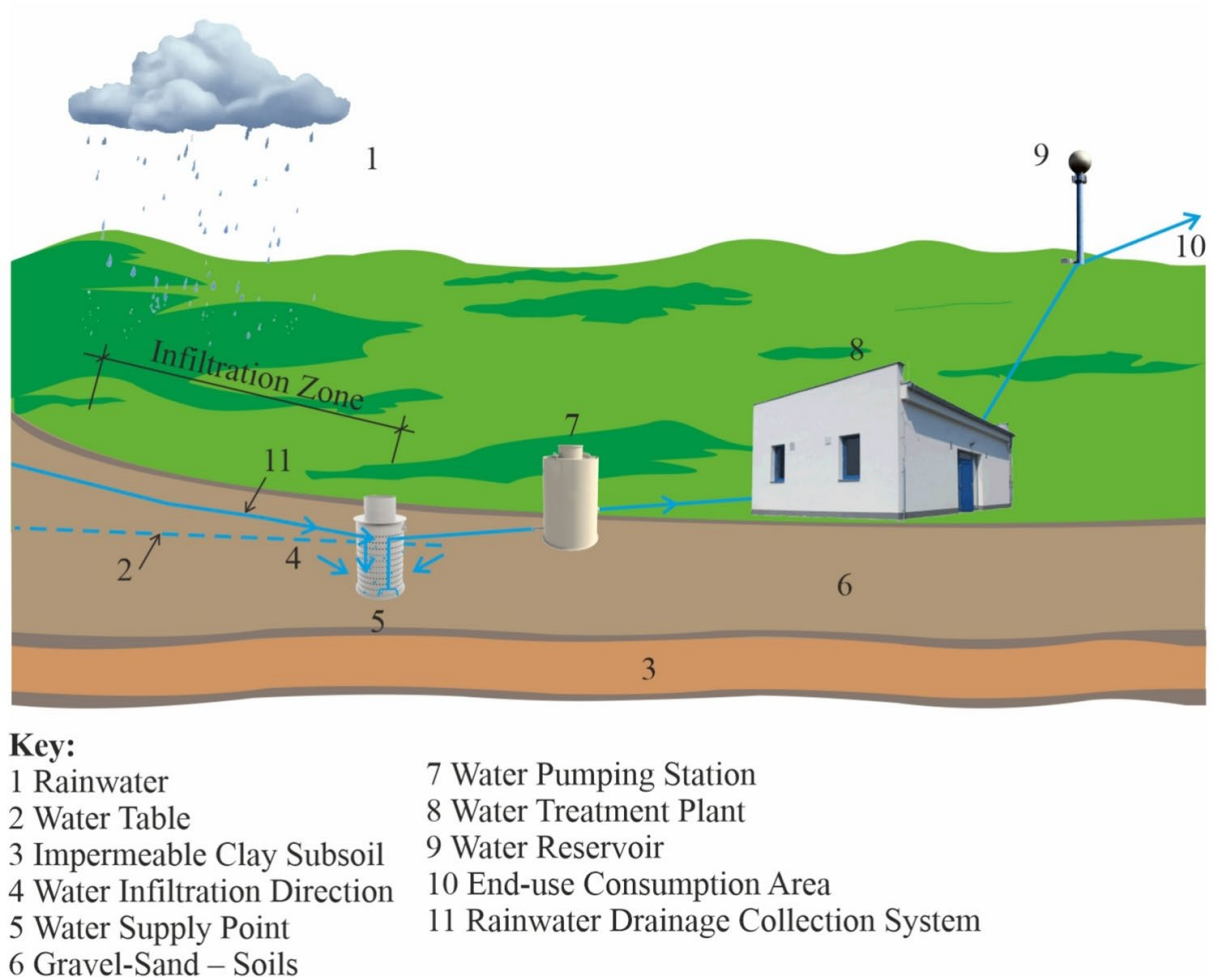
Controlled permeation in the end-use consumption area/authors/

**Fig. 4:**
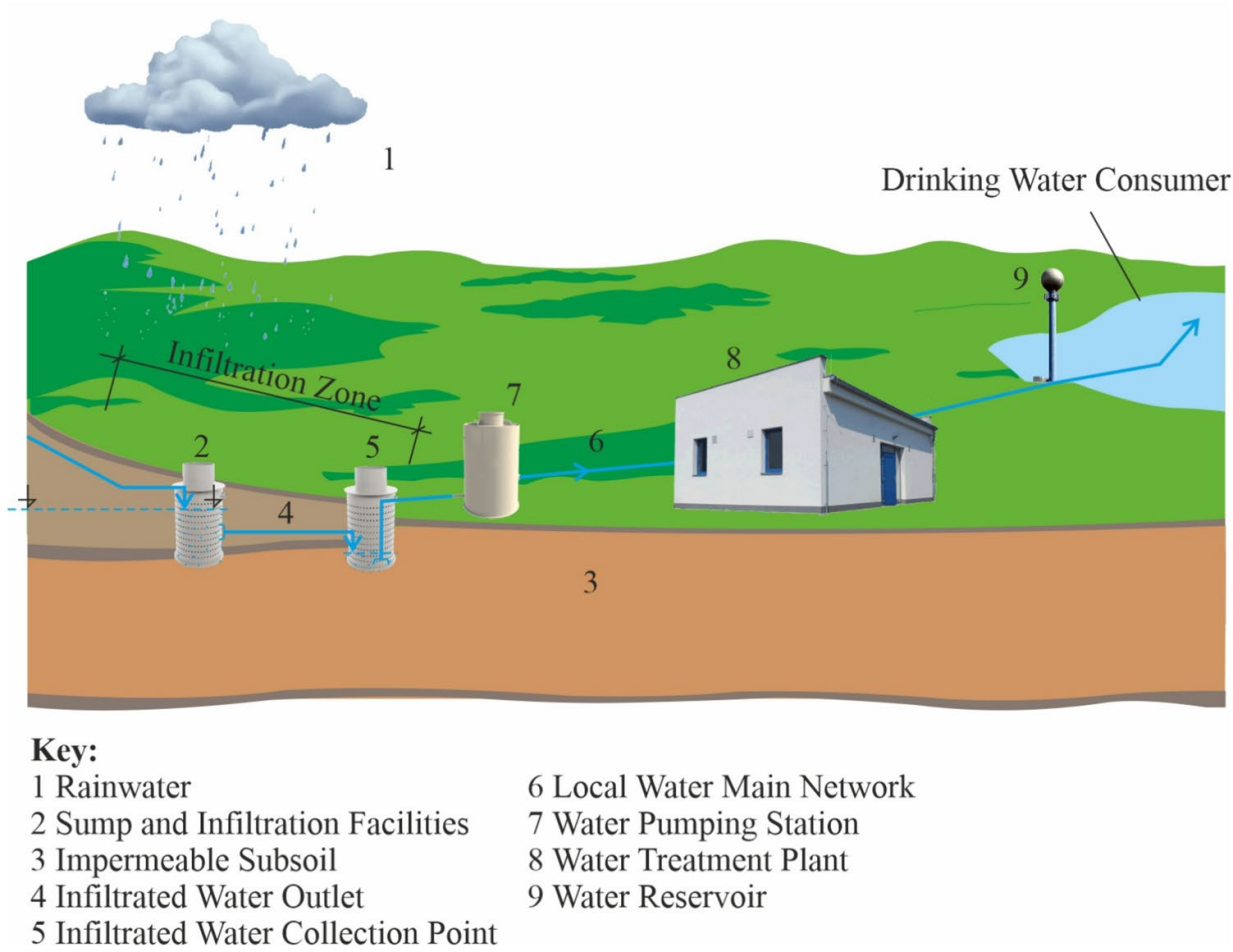
Exploitation outside the end-use consumption area/authors/

The above mentioned basic ways of water volume growth intended for water supply management purposes can be implemented mainly through the means of two basic forms and ways of infiltrated water utilization. The whole process is depicted in Figs. 3 and 4.

Both above shown variants follow the same goal in practice, i.e., to improve the rainfall utilization not only for water supply management purposes but now also in the field of general precipitation management in every country of the world.

Increased attention has been paid to the issue of rainwater retention in the Czech Republic for some time, especially due to the lack of other ways of providing high quality supplies of raw water suitable for its processing into drinking water. The methods shown in Figs. 3 and 4 especially include the construction of water reservoirs with high degree of protection from secondary contamination through the creation of water source protection zones. The primary options of achieving the specified protection are mentioned in the following chapter.

### Protection of surface water and groundwater from contamination

**Fig. 5:**
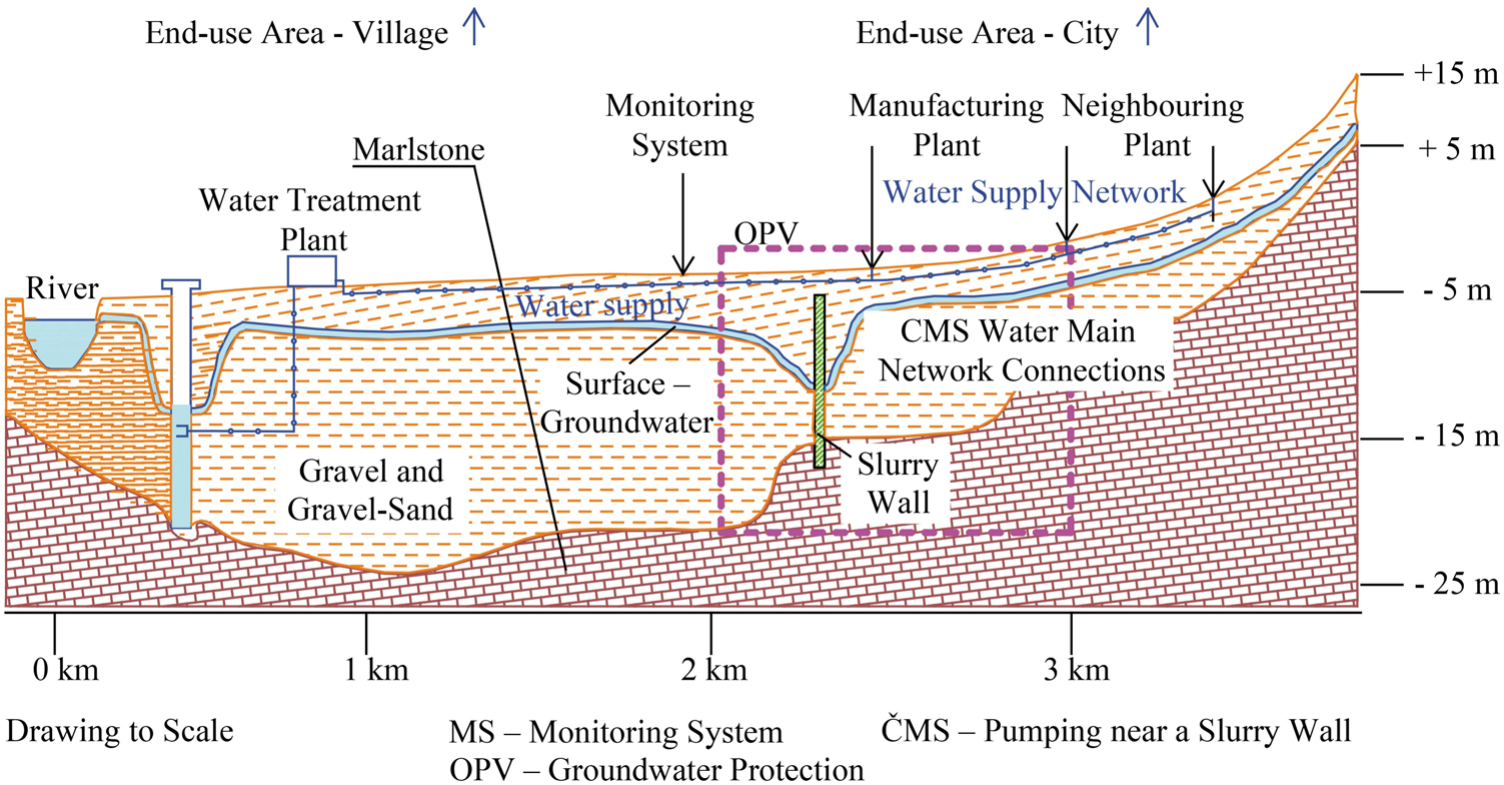
Diagram of groundwater protection using a slurry wall/authors/

As indicated in the previous chapters, the danger of contamination of surface water as well as groundwater is increasing. The cause of this situation is the development of human knowledge and the usage of new materials and technologies of their processing with deficiencies in the field of prevention, as well as the relatively frequent occurrence of new accidents caused by old ecological burdens which arise from the use of the original technological materials beyond their service time on the time axis of their service life (Yang and Liu 2020). The most dangerous types of old ecological burdens are caused by unregistered dumps of chemical substances in industrial agglomerations after the end of service life of the original chemical substance containers and the subsequent contamination of soil layers and groundwater. In fact, the only feasible protection is the construction of a slurry wall around the entire source of contamination, see Fig. 5, with diligent monitoring of contaminated waters and observing hydraulic conditions in the wider area around the place of environment contamination.

One of the most serious problems concerning surface water and groundwater throughout the whole twenty-first century will be the change of dilution ratio of these waters as a consequence of ongoing global climatic change. The already high environmental material burden of aquatic ecosystems caused by noxious and hazardous substances will obviously be rising in the future. This poses a threat not only to the current fauna and flora but also to the surface and ground sources of drinking water.

Under the conditions of European Union countries, it is not possible to utilize any raw water but only the water which meets the requirements stated in the EU Council Directives No. 75/440 EEC regarding the quality of surface water used for drinking water abstraction/the directive lost its effect in 2007/(Council Directive 75/440/EEC 1975), the Directive of the EU Parliament and Council 2000/60/ES from 23 October 2000, which sets the scope for Community water policy (European Union 2020) and the EU Council Directive No. 79/869 EEC regarding the methods of measuring and abstraction frequency of surface water (Council Directive 79/869/EEC 1979). From the above stated, it is apparent that maintaining the quality of raw water in aquatic ecosystems within the threshold values in categories A1, A2, A3, specifying the determination of the average index of water treatability in water supply management processes and ways of their technological processing into drinking water, will be continuously more demanding.

The Czech Republic has been fulfilling the required criteria for decades and permanently focuses on the long-term sustainability of this situation. Not only the sufficient legislative environment but also the advanced infrastructure and monitoring systems, which are able to issue a warning of impending danger in time, help to achieve this aim. Even with these successes achieved in the Czech Republic, a lasting task is to improve the current systems in accordance with new scientific knowledge in this field and the technological inventions in the field of monitoring technologies.

### Prevention of pollution and maintaining long-term sustainability of aquatic ecosystems

**Fig. 6:**
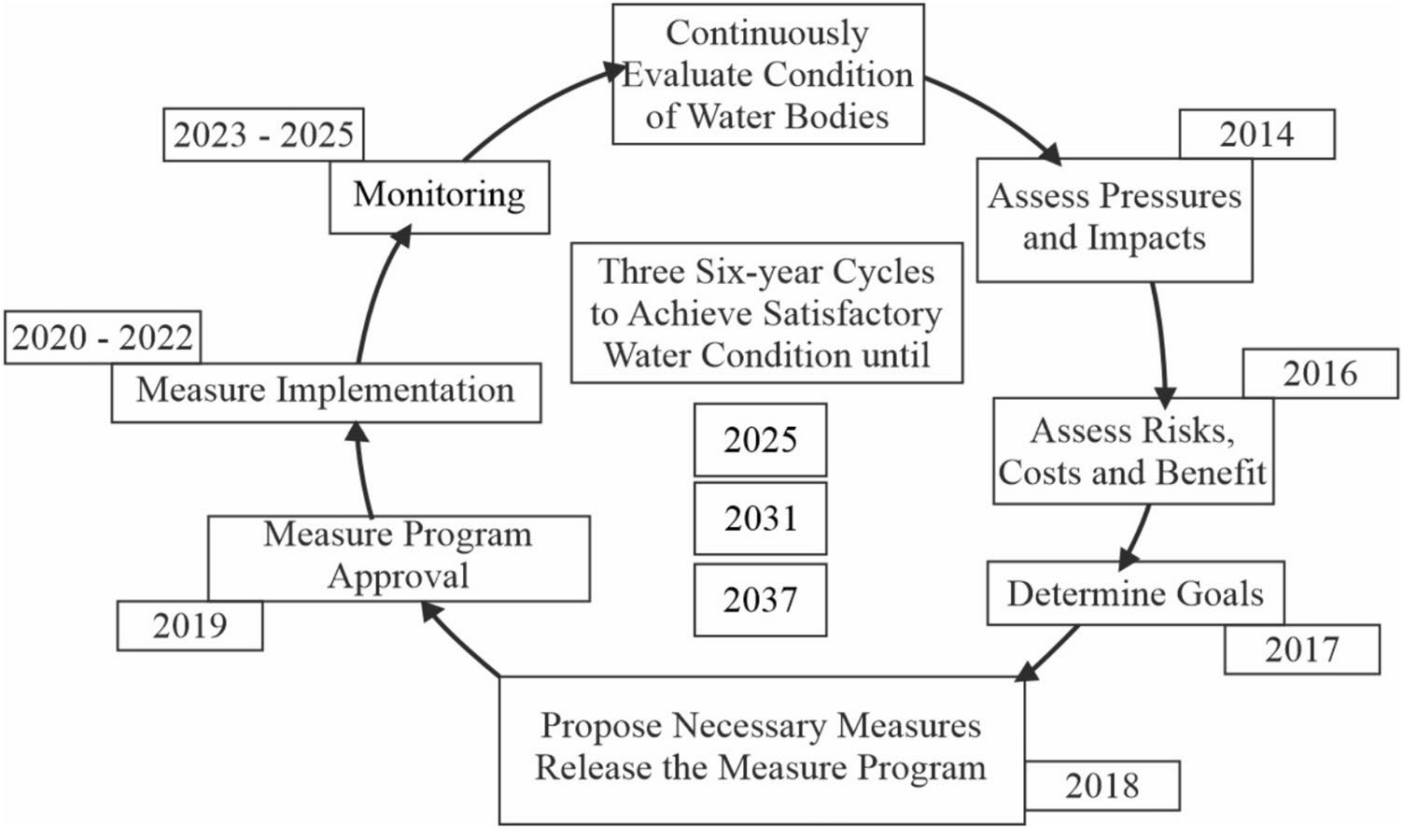
Process of significant improvement of European waters

The natural environment and aquatic ecosystems do not have or respect any artificial boundaries of regions and states. When tackling these issues, for example in water resource management, it is essential to consider Europe as one hydrological unit and to solve its issues as shown in Fig. 6.

**Fig. 7:**
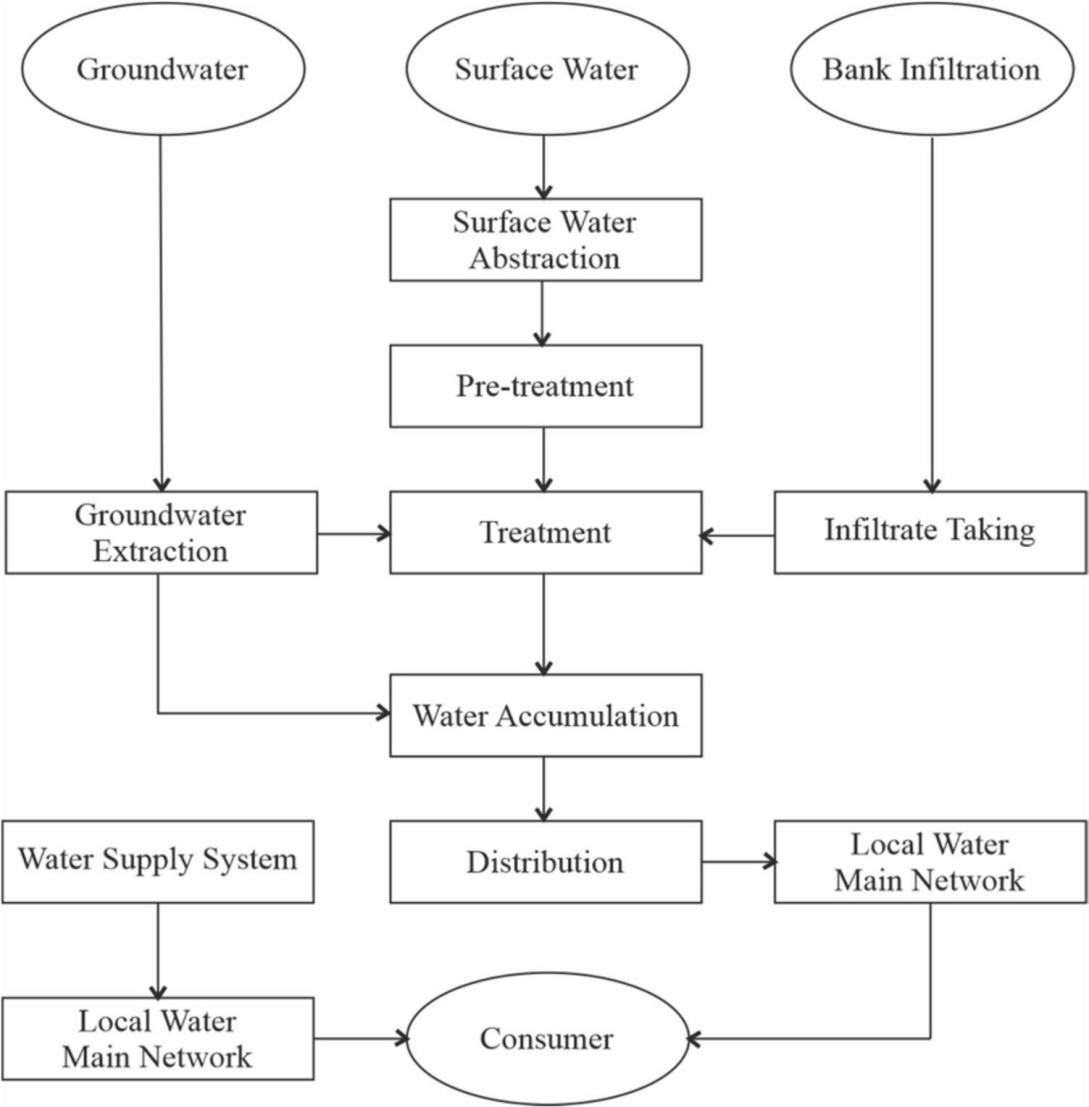
Diagram of water transfer from a natural resource to a consumer/authors/

Waters and water environments cannot be efficiently protected when segmented, in individual countries, but only globally. The smallest unit of efficient protection is the drainage basin in question. The way of aquatic ecosystem protection in European Union countries is based on the previous statement. The previous figure is a simplified illustration of how to achieve the required protection. In the strict sense of the word, the waters used in the treatment process for turning them into drinking, utility, and other technological waters are depicted in Fig. 7.

### FMEA method

**Fig. 8:**
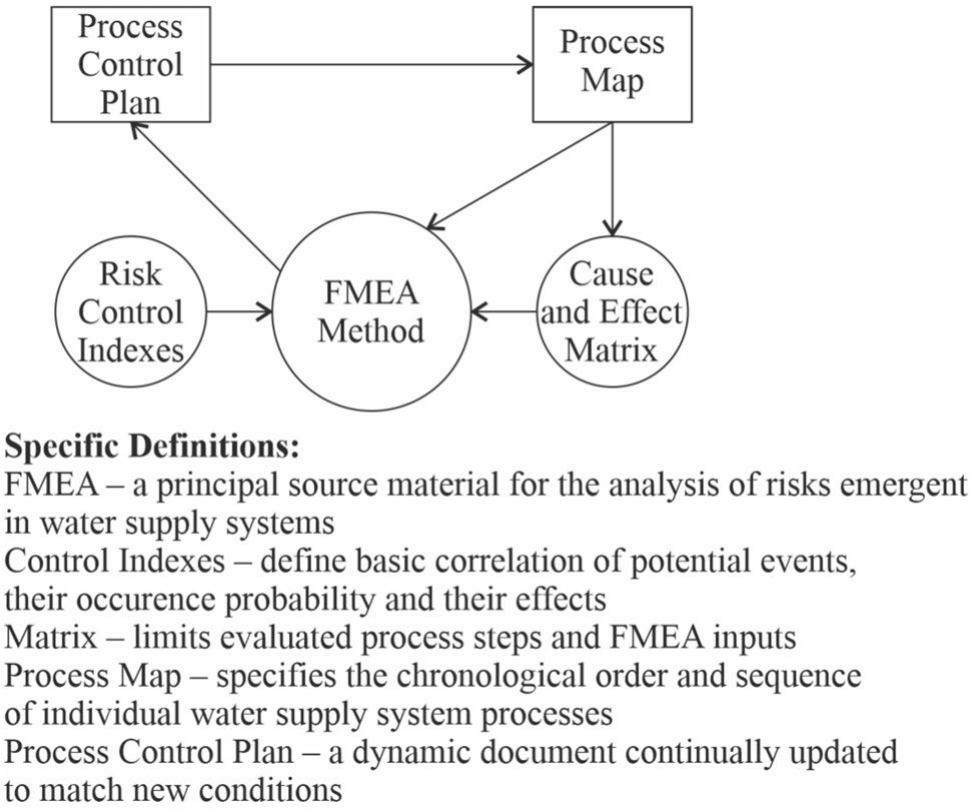
Mutual connections of operational risks and relations in risk analysis/authors/

The complexity of water treatment processing it into drinking or technological water always depends on the purity of the water abstracted from an underground or surface source. When dealing with threatened aquatic ecosystems or those with already contaminated aquiferous soil layers, it is essential to prepare for a solution by using suitable methods. It is optimal for these systems to use the FMEA method and the control index method, which create sufficient space for an analysis. An overview of mutual connections of operational risks and relationships in risk analysis is described in Fig. 8.

The FMEA method, when appropriately used, has two primary goals, in which the following questions are to be answered:

Finding a structured way to the goal.

- Identification of ways in which the analysis may fail completely or partially.
- Estimation of risks of particular cause of specified failure.
- Determination of priorities leading to goal achievement, which may decrease the danger of failure.
- Elaboration of a control plan which enables systematic avoidance of errors in the solving project.

The achievement of the determined goal is significantly supported by respecting the above described basic stimuli, and possibly other aspects, in relation to the solution scope. At this point, it also indicates what the purpose of seeking this way is.

Purpose of seeking this way:

- The purpose of the used FMEA method is the identification of possible failure regarding the issues in question within operational conditions and the analysis of their effect relevancy.
- Defining mutual dependencies.
- Ordering of alternative errors.
- Precautionary measures against error occurrence.

If needed, the FMEA method includes the elaboration of a cause and effect matrix, which is preceded by risk control indexes of the environment or aquatic ecosystem in question. One of the important aspects of the issue in question is its simplicity and understandability. It is essential to presume the fact that the unintelligibility of the control plan and other measures generally leads not only to its misunderstanding, but at the same time, it decreases the expected outputs of the entire solution of the issues in question.

A wide range of methods is used for analyses and solutions of issues in question in the Czech Republic. The use of a particular method or the development of a new one always depends on the conditions and the kind of aquatic ecosystem, its inclusion into the wider scope of environmental protection in a given drainage basin, as well as the possibilities of practical implementation. The most complex cases are in former industrial agglomerations of chemical and power system fields. What makes the conditions in the Czech Republic complicated is the current lack of surface water suitable for its treatment turning it into drinkable water while maintaining at least a basic balance between the outtake and the sources of water from rain and snow precipitations.

The FMEA method was used for the initial analysis. In this part, the analysis of the above mentioned risks was practically applied. For each risk, the significance, probability of occurrence and probability of detection were assessed and the resulting risk priority numbers (RPN) were calculated according to this assessment. The process and results of the analysis were consulted in an expert team, which, in addition to the three authors of the study, consisted of experts from the Vltava River Basin, Elbe River Basin, Morava River Basin—state enterprise and the Czech Hydrometeorological Institute. Thus, a representative group of experts from practice and academia was assembled to evaluate the individual criteria of the FMEA analysis. The results are interpreted in Table 2. Evaluation criteria are described in Table 3, Table 4, and Table 5.

**Table 2:** Application of the FMEA Method to the conditions of the Czech Republic according to the authors

Process identification:								
Potential risk	Possible risk consequences	Possible cause	Current status					Recommended measures
			Existing measures	Significance	Occurrence	Detection	RPN	
1. Contamination with noxious and hazardous substances	Deterioration of drinking water quality indices, impact on aquatic ecosystems	Accidents involving crude oil substance spillage old ecological burdens (uranium ore processing MAPE Mydlovary, former oils and mazut manufacturing in Ostrava)	Monitoring, slurry wall construction, hydraulic barrier construction	9	9	2	162	Increased monitoring and sample collection. Installation of stable water baffles
2. Groundwater yield decrease	Insufficient source capacity for drinking water supply need	Long-lasting drought, poor economical use of water in the region (areas of South Moravia – Hodonín, South Bohemia)	Retention basins, source water protection area	6	7	4	168	Change in the usage of lands surrounding the water source. Artificial water infiltration into aquifers
3. Surface water yield decrease	Insufficient source capacity of drinking and service waters for civil and infrastructure supply needs; impact on aquatic ecosystems	Long-lasting drought, poor economical use of water in the region	Economical and sustainable agriculture in the region	8	7	1	56	Building retention basins
4. Pollution of drinking water with wastewater	Impact on the ecosystem and water quality	Insufficient or ineffective wastewater treatment, accidents on sewage systems(floods in Moravia in 1997 – city Ostrava, 2002 and 2013 city České Budějovice)	Increase in wastewater treatment effectivity	7	5	2	70	Protection of sewage treatment plants from floods
5. Pollution as a result of atmospheric emissions	Contamination of surface water, groundwater, and soil	Rainfall in major industrial regions (Ostrava, Ústí)	Building separate sewerages in municipalities (separately for sewage and separately for rainwater)	4	3	7	84	Decrease of emissions
6. Agricultural pollution	Contamination of aquatic ecosystems and negative impacts on biodiversity	Improper and excessive usage of fertilizers, pesticides and chemical substances in agriculture	Inspection of economical water management in source water protection areas, inspection of adherence to the measures implemented in source water protection areas	3	4	4	48	Implementation of sustainable and ecological agriculture measures

**Table 3:** Evaluation criteria—significance (impact)

Significance (impact)	
Barely noticeable	= 1
Insignificant	= 2 ÷ 3
Moderate	= 4 ÷ 6
Severe	= 7 ÷ 8
Critical	= 9 ÷ 10

**Table 4:** Evaluation criteria—likelihood of a problem occurrence

Likelihood of a problem occurrence	
Unlikely	= 1
Very low	= 2 ÷ 3
Low	= 4 ÷ 6
Medium	= 7 ÷ 8
High	= 9 ÷ 10

**Table 5:** Evaluation criteria—likelihood of detection (prior to output)

Likelihood of detection (prior to output)	
High	= 1
Medium	= 2 ÷ 5 RPN = significance*occurrence*detection
Low	= 6 ÷ 8 (risk priority number)
Very low	= 9
Unlikely	= 10

## Discussion

The results of the FMEA analysis—for the water source factor, the most significant risk was the reduction of groundwater yield (RPN 168), which may result in insufficient capacity of the source for supplying drinking and fire water to the population, and contamination with hazardous and dangerous substances (RPN 162), resulting in deterioration of drinking water quality indicators and significant impact on aquatic ecosystems.

These threats include in particular the following:

- Contamination of surface and groundwater
- Temporary decommissioning of drinking water sources
- Serious environmental damage to fauna and flora in the catchment area

Both of these factors can be assessed as a serious risk with the possibility of a partial threat to the water supply system for the population and to the smooth operation of the infrastructure in the area to be supplied. Another significant risk is the contamination of surface and ground water and soil due to atmospheric emissions (RPN 84) This factor is significant in areas with high industrial activity, for example, in the Czech Republic in northern Moravia and northern Bohemia. Ecosystem and water quality are affected by the risk of pollution of water sources by waste water (RPN 70).

With the coming climate change, not only an increase in the number of natural emergencies can be expected but also an increase in the intensity of secondary effects of anthropogenic emergencies in the affected area. Scientific knowledge is of fundamental importance in furthering the process of long-term sustainability of the territory and its environment. Together with the technical development of the industrial base, they have the potential to create defence systems that reduce current security risks. However, it is always advisable for science and technology to provide other stimuli which, following initial analyses, can be part of the development of security. One of these avenues, may be to address the issue below:

- A change in approach to address past ecological failures in the sustainability of rainfall in areas at risk of hydrological drought
- Ways of deactivating large parts of land reclamation structures
- The development of new types of monitoring systems for the effects of climate change on surface and groundwater supplies, particularly in areas at risk of meteorological drought

The above and other research and the search for new ways, based on scientific risk analysis while maintaining a link to the possibilities of technological development, can already in a relatively short period of time mitigate a significant part of security threats and at the same time prevent the emergence of new threats arising in individual regions of the world.

Worldwide pollution of aquatic ecosystems has been gradually increasing for centuries. It cannot be assumed that there will be some change in the tendency threatening the original nature balance (United Nations World Water Assessment Programme 2015) without using effective countermeasures. A positive value in this field seems to be the possibility to efficiently resist the set tendencies with the help of the latest scientific knowledge in the fields of chemistry, material engineering, and safety engineering.

The climatic conditions of individual states or regions are not an unchanging quantity—they tend to change over time from natural, and currently also anthropogenic causes. Since the beginning of the twenty-first century, one of these changes has been taking place worldwide, which, among other things, will also affect the aquatic ecosystems of the Czech (Brumar et al. 2018; Dušek 2015). Development trends suggest that natural volumes of surface and groundwater are most likely to be significantly reduced in the coming years and decades. In many regions in the Czech Republic, smaller recipients of watercourses and groundwater reserves will be significantly endangered (Walmsley et al. 2020). If artificial water accumulations can be operatively managed and adequately replenished depending on snow and rainfall, then running water and groundwater will be at risk of at least a periodic shortage. This is concurrent with the need to observe sufficient protection against pollution and increasing the concentration of dangerous substances in water and in the entire field of water resource management. However, a large part of this type of aquatic ecosystem is used for water supply purposes and for fire safety of an area, and in many cases as the only source of fire water.

Modern science and scientific knowledge of processes and their interconnection enable us not only to study the positive and negative aspects of natural processes, but also to mitigate negative impacts on the environment using the appropriately selected methods. In view of the fact that aquatic ecosystems are one of the most important aspects of all processes on Earth, it is essential to pay them maximum attention not only in scientific circles but also among experts and the public globally. With the global drought, which is already affecting a third of the world’s populated areas, every further deterioration of the current situation is a serious warning, which must not be underestimated or ignored. One of the essential steps is threat assessment.

Threat assessment of aquatic ecosystem contamination is a complex process (Rehak et al. 2019; Brehovská et al. 2015) which requires the integration of various factors and methodologies. The key steps and factors which should be included while assessing threats are as follows:

- Identification of potential contaminators: Identification of pollution sources, such as industrial plants, agriculture, municipal sewage waters, surface runoff from urbanized areas etc., is the crucial first step. This also includes the identification of chemicals and substances which may be released into aquatic ecosystems.
- The determination of target ecological and human values: The determination of values we want to protect, such as the biodiversity of aquatic ecosystems, quality of water intended for drinking, recreation and other economic or social uses, is essential for assessing the importance of contamination threats.
- Exposition analysis: An assessment of how aquatic ecosystems are exposed to various types of pollution including the quantification of the amount and type of the pollutants entering them and their frequency.
- Ecosystem sensitivity assessment: The evaluation of the sensitivity of aquatic ecosystems to various types of pollution and their reaction to contamination. This may include studies on the effect of pollution on biodiversity, ecosystem functions, and health of aquatic organisms population.
- Risk assessment: The combination of the exposition and sensitivity of ecosystems allows to assess the risk of aquatic ecosystems contamination. This includes the evaluation of the risk occurrence probability and the severity of its impacts on ecological and human values.
- Planning of measures: Based on the risk assessment, it is necessary to work out the plans for decreasing the risk of contamination and the protection of aquatic ecosystems. This may include measures taken to check and regulate the contamination sources, to improve the infrastructure for waste management, to monitor the quality of water and to implement the measures for climate change adaptation.

Climate change is relevant in water management with increasing extreme events affecting landscape stability. Threats of contaminated drinking water sources, sustainable, and efficient water management are therefore key to adapting to climate change. European policy increasingly makes sustainable practices in response to climate change and a societal priority, requiring the need for agroecological approaches, efficient water use and water management reform. Research underlines the importance of maintaining the water balance and adapting water management to regional climate risks (Bednář et al. 2025).

While assessing the threats of aquatic ecosystems contamination, the above-mentioned steps should be carried out in cooperation with a multidisciplinary team of experts in the fields of ecology, hydrology, chemistry, public health and other relevant fields. To prevent the development of threats with an increased risk of aquatic ecosystems contamination caused by climate change, it is important to monitor the quality of aquatic ecosystems, to enhance pollution precautions, and to implement climate change adaptation measures, such as the improvement of infrastructure of rainwater management and water source protection. Moreover, it is essential to regulate and reduce the emissions of pollutants so that their influence on aquatic ecosystems is minimized.

From discussions taking place among Czech water management specialists, experts and the media dealing with this subject matter, it is apparent that all involved parties are concerned about the decrease of rain precipitation in certain regions of the Czech Republic, and especially the decrease of snow precipitation and their amount within the Czech Republic. If the irregularity and intensity of rainfall can be relatively easily substituted by the construction of hydraulic structures which retain rainwater, then snowfall has the dominant influence on the yield of groundwater designated and used particularly for drinking purposes, which currently serves as the only source of drinking water for citizens and for the local infrastructure of the region in question.

The study reveals complex relationships affecting threats of contamination of drinking water sources and preventive enhancement of protection efforts of these sources their protection. The diversity of impacts of climate change may increase the risk of aquatic ecosystems contamination in various ways from precipitation changes through rising water temperatures and increased frequency of extreme events and others. The interaction between risks and societal resilience at the local and global levels may manifest differently, which underlines the importance of combining different methods to understand complex socio-economic phenomena (Locatelli et al. 2015) and the need for holistic approaches to understanding proper water management and minimizing threats of contamination of drinking water sources in the landscape (Petrovič et al. 2017).

The diversity of attitudes with impacts on prevention and enhancement of protection of drinking water sources can be shown on the example of farmers in the Czech Republic. This approach shows a significant diversity of attitudes towards sustainable agriculture and water management. Organic farmers consistently demonstrate more positive attitudes towards the environment and water management than conventional farmers, with integrated farmers occupying an intermediate position. This is consistent with previous research (Bourceret et al. 2023) and suggests a gradual transition towards sustainability influenced by a combination of economic incentives, educational attainment, and deeply held personal values.

The minimization of threats of aquatic ecosystems contamination in the Czech Republic requires a complex approach, which includes the regulation of industrial emissions, the improvement of wastewater treatment, the support of sustainable agricultural practices, the protection of natural biotopes and water sources, and the support for the education of the public on the importance of aquatic ecosystems protection. Authors should discuss the results and how they can be interpreted from the perspective of previous studies and of the working hypotheses. The findings and their implications should be discussed in the broadest context possible. Future research directions may also be highlighted.

## Data Availability

The qualitative data collected is directly available in the core of this article.
